# The dosimetric and radiobiological effects of rotational errors in breast cancer radiotherapy

**DOI:** 10.1002/acm2.70303

**Published:** 2025-10-15

**Authors:** Denghong Liu, Quan Zhong, Ya Wang, Pan Gong, Xiangbin Zhang, Jialu Lai, Shichao Wang, Shoupeng Liu, Zhonghua Deng, Konglong Shen, Bin Du, Ruilin Peng, Renming Zhong

**Affiliations:** ^1^ Radiotherapy Physics & Technology Center Cancer Center West China Hospital Sichuan University Chengdu People's Republic of China; ^2^ Department of Oncology Fifth People's Hospital Affiliated to Chengdu University of Traditional Chinese Medicine Chengdu People's Republic of China

**Keywords:** breast cancer radiotherapy, dosimetry, normal tissue complication probability, rotational errors, tumor control probability

## Abstract

**Purpose:**

This study aimed to assess the dosimetric and radiobiological consequences of rotational errors in breast cancer radiotherapy.

**Methods:**

A retrospective analysis involving 80 breast cancer patients was performed. We simulated 124 rotational scenarios across three axes (yaw, pitch, and roll) to generate rotated dose distributions, then evaluated their dosimetric effects alongside tumor control probability (TCP) and normal tissue complication probability (NTCP) for the target and organs at risk (OARs).

**Results:**

Rotational errors caused significant dose deviations. The D95 for PTV, heart, ipsilateral lung, contralateral breast, and left anterior descending artery (LAD_V40_) in rotated dose distributions showed statistical significance compared to the original dose (*p* < 0.05), except for LAD_V40_ in the whole‐breast radiation therapy (WBRT) _left4005_ cohort (*p* = 0.058). LAD_V40_ and PTV_sc_ were primarily affected in left‐sided breast cancer, while PTV_sc_ and PTV_cw_ were predominantly affected in right‐sided breast cancer. TCP decreased from 0.91 to 0.81 in the WBRT_4005_ cohort, from 0.95 to 0.70 in the WBRT_5000_ cohort, and from 0.87 to 0.23 in postmastectomy radiation therapy (PMRT)_5000_ cohort. NTCP for the heart increased from 2.08 × 10^−14^ to 5.78 × 10^−10^, and NTCP for the lung increased from 1.82 × 10^−3^ to 1.37 × 10^−2^. When single‐axis rotations were involved, the pitch direction was more likely to cause dose deviations. For multi‐axis (≥2) rotations, opposite‐direction yaw and pitch rotations in left‐sided breast cancer and same‐direction yaw and pitch rotations in right‐sided breast were more prone to exceeding dose limits.

**Conclusion:**

The dosimetric and radiobiological analysis of rotational errors demonstrated that rotational errors (particularly in pitch and yaw) induce significant dosimetric deviations, compromising tumor control probability and increasing normal tissue complication probability. It identifies rotational axes requiring correction during multi‐axis rotations and provides recommendations for prioritizing targets versus organs at risk during image‐guided registration. Implementing these corrections and prioritization strategies is essential for optimizing therapeutic efficacy in breast radiotherapy.

## 1 INTRODUCTION

Since 2021, breast cancer has ranked as the most common female malignancy worldwide.^1^ Postoperative adjuvant radiotherapy is a critical component of comprehensive breast cancer treatment, playing a pivotal role in improving long‐term survival and treatment outcomes.^2^, ^3^ For early‐stage breast cancer patients who have undergone breast‐conserving surgery, whole‐breast radiation therapy (WBRT) remains the standard adjuvant treatment.^4^, ^5^, ^6^ In intermediate‐ to high‐risk patients receiving postmastectomy radiation therapy (PMRT), irradiation of the chest wall and supraclavicular lymph nodes is indispensable.^7^, ^8^, ^9^

In breast cancer radiotherapy, precise immobilization is fundamental to ensuring treatment efficacy and safety. Common immobilization techniques include breast‐specific brackets,^10^ vacuum pads,^11^, ^12^ and thermoplastic masks,^13^ which are designed to provide personalized support for patients. However, despite the use of these immobilization methods, translational and rotational errors are inevitable, compromising dose delivery accuracy and potentially affecting treatment outcomes.^14^, ^15^, ^16^, ^17^

In clinical practice, imaging guidance is routinely employed to identify and correct left‐right (L‐R), superior‐inferior (S‐I), and anterior‐posterior (A‐P) translational errors, as well as rotational errors (pitch, yaw, and roll) using a six‐degrees‐of‐freedom (6‐DOF) couch.^18^, ^19^, ^20^, ^21^ However, owing to the unavailability or operational constraints of the 6‐DOF couch, only translational errors are typically corrected. During treatment, if rotational deviations exceed 3°—regardless of the axis—therapists will often reposition the patients; in contrast, deviations below 3° are typically tolerated based on clinical experience. This experience‐based approach lacks standardized guidelines and may even lead to excessive radiation exposure due to suboptimal repositioning.^22^ Specifically, there is a critical need to investigate which rotational axes most significantly affect dose distribution to the target and organs at risk (OARs), as well as their impacts on tumor control probability (TCP) and normal tissue complication probability (NTCP).

Thus, the objective of this study was to evaluate the dosimetric and radiobiological impacts of rotational errors in breast cancer radiotherapy on the target, heart (specifically the left anterior descending artery, LAD), and ipsilateral lung.

## 2 METHODS

### 2.1 Patients, CT simulation, and structure definition

Eighty breast cancer patients treated with Synergy and VersaHD accelerators (Elekta, Stockholm, Sweden) at our center between June 2023 and June 2024 were randomly selected for retrospective analysis. Patient characteristics are presented in Table 1. This study was approved by the Ethics Committee of West China School of Medicine, Sichuan University (Approval No. 2025227).

**TABLE 1:** Patient characteristics.

Characteristics	No. (%)
Left‐sided breast cancer	
WBRT (cases)	20
WBRT_4005_	10
WBRT_5000_	10
Age (years)	
Median (range)	40 (32–51)
Weight (kg)	
Median (range)	51 (43–66)
Stage	I–II
PMRT (cases)	
PMRT_5000_	20
Age (years)	
Median (range)	55 (44–64)
Weight (kg)	
Median (range)	51 (45–57)
Stage	II–III
Right‐sided breast cancer	
WBRT (cases)	20
WBRT_4005_	10
WBRT_5000_	10
Age (years)	
Median (range)	41 (30–53)
Weight (kg)	
Median (range)	52 (46–60)
Stage	I–II
PMRT (cases)	
PMRT_5000_	20
Age (years)	
Median (range)	56 (48–63)
Weight (kg)	
Median (range)	51 (43–58)
Stage	II–III

*Note*: WBRT_4005_ indicates a prescribed dose of 4005 cGy of WBRT, WBRT_5000_ indicates a prescribed dose of 5000 cGy of WBRT, PMRT_5000_ indicates a prescribed dose of 5000 cGy of PMRT.

Abbreviations: WBRT, Whole Breast Radiation Therapy; PMRT, Postmastectomy Radiation Therapy.

All patients were positioned supine on a 15° wedge plate with a breast vacuum bag (Klarity Medical, Shenzhen, China) and underwent simulation using a GE Revolution CT scanner (GE Medical Systems, Milwaukee, WI). The CT scans extended from the chin to the lower liver border with a slice thickness of 3 mm. Scanned images were transferred to the treatment planning system for contouring. Radiation oncologists delineated the clinical target volume (CTV) and organs at risk (OARs) on the images following the ESTRO guidelines.^23^ The planning target volume (PTV) was generated by adding a 5 mm margin to the CTV.

### 2.2 Treatment planning

Experienced physicists designed the original treatment plans on planning CT images using RayStation 9A (RaySearch Laboratories, Stockholm, Sweden), with oncologist approval prior to treatment. All treatment plans utilized 6 MV photon beams, and dose calculations were performed using a collapsed cone algorithm. To avoid overdosing the contralateral breast, lung, and heart, beam arrangements were approximately 210°–60° for right‐sided breast cancer and 280°–160° for left‐sided breast cancer.

### 2.3 Acquisition of rotational errors

**FIGURE 1:**
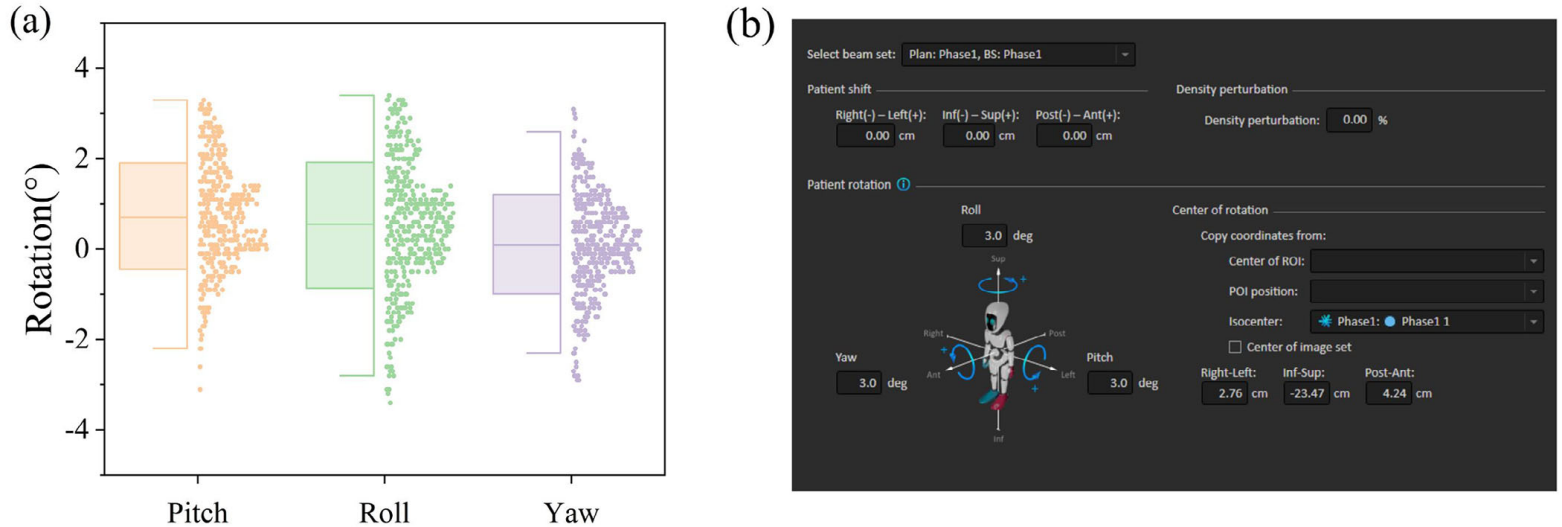
Rotational errors and calculated rotated dose distribution for a combined 3° rotation in yaw, pitch, and roll. (a) Rotational errors. (b) Calculated rotated dose distribution for a 3° rotation in yaw, pitch, and roll.

Rotational errors were retrospectively collected from 80 patients at our center. Detailed records of rotational errors were obtained during the first three treatment sessions for each patient, with additional weekly recordings conducted over six subsequent sessions. As illustrated in Figure 1a, patient rotational errors predominantly clustered between ± 1° and ± 2° across the pitch, roll, and yaw axes, with maximum errors reaching approximately ± 3°. This finding is consistent with the study by Shen et al.^11^

### 2.4 Calculation of the rotated dose

As outlined in Section 3, the immobilization methods used at our center result in a maximum rotational error of approximately 3°. To model this, the original plans were imported into RaySearch 12A (RaySearch Laboratories, Stockholm, Sweden), and rotated dose distributions were calculated by simulating 124 combinations of rotations across the three axes (yaw, pitch, and roll). The initial rotational state was defined as (0°, 0°, 0°). As depicted in Figure 1b, the rotated dose distribution was calculated for a combined 3° rotation in yaw, pitch, and roll. Notably, 1° rotations were excluded from the simulations. Prior research has established that rotational errors below 2° are generally considered acceptable.^24^

### 2.5 Identification of rotational scenarios that exceed dose limits

**FIGURE 2:**
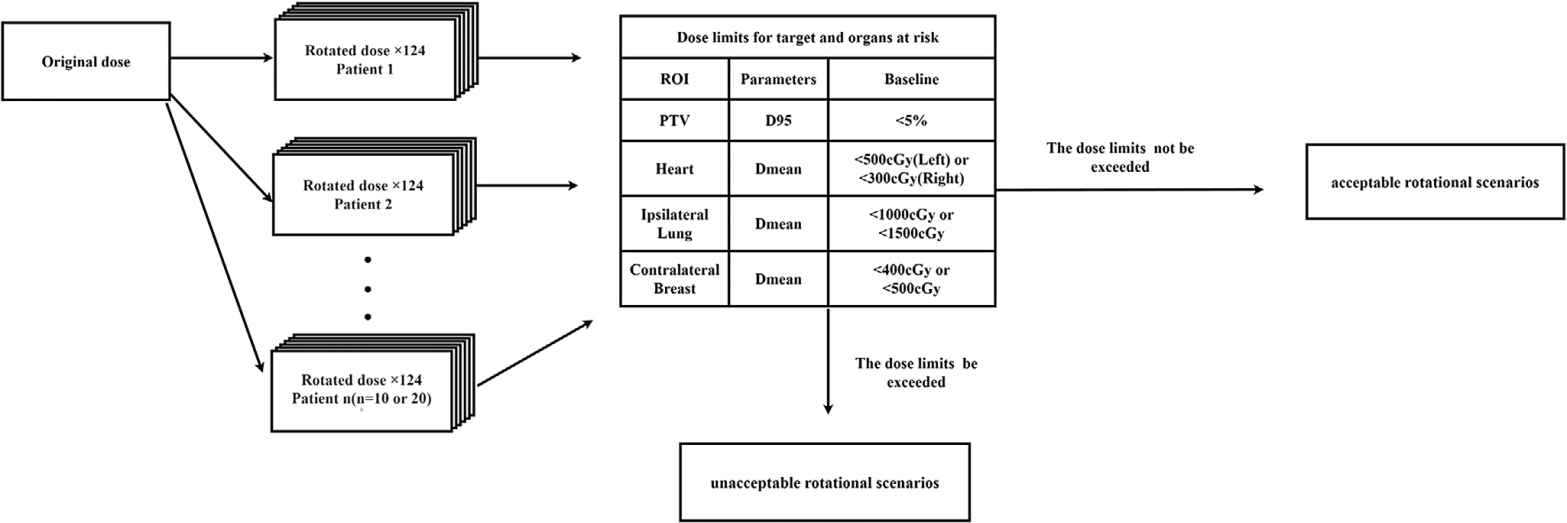
Flowchart for identifying rotational scenarios that exceed dose limits. ROI, region of interest.

According to the ICRU83 report^25^ and the guidelines for postoperative radiotherapy target delineation and planning design for breast cancer,^26^ 124 rotational scenarios were simulated for each patient. The simulations evaluated whether each scenario met two criteria: (1) dose deviation within 5% of the planned dose, and (2) compliance with all predefined dose‐volume constraints for OARs. Scenarios satisfying these criteria were classified as having no effect on the target and OARs. Conversely, non‐compliant scenarios were flagged for causing dose discrepancies in target and OARs, enabling filtration of scenarios exceeding predefined dose limits and counting the number of patients with such scenarios. The screening methodology is outlined in the flowchart shown in Figure 2.

### 2.6 TCP and NTCP calculation

The TCP and NTCP were computed employing the linear‐quadratic Poisson model and the Lyman‐Kutcher‐Burman model, respectively. These calculations utilized the pyradiobiology package^27^, ^28^ and incorporated the physical dose data exported from the RayStation planning system. The radiobiological parameters and endpoints used for TCP/NTCP calculations were specified in previous studies^29^, ^30^, ^31^, ^32^ and listed in Table 2.

**TABLE 2:** Set of parameters of TCP/NTCP.

ROI	TCD_50_ (Gy)	TD_50_ (Gy)	*γ*	*α*/*β*	*m*	*n*	motes
PTV	30.89		1.3	4			Tumor control model derived from single‐institution data of T1+T2 raditiontherapy
PTV	39.3		1.7	4			Tumor control model derived from multi‐institution data of adjuvant radiation therapy
Heart		48		3	0.1	0.35	Endpoints: pericarditis
Lung		30.8		3	0.18	0.87	Endpoints: pneumonitis

### 2.7 Statistical analysis

Statistical analyses were performed using SPSS software (version 26.0; IBM, Armonk, NY, USA). The Shapiro‐Wilk test confirmed that all parameters were normally distributed, while the Homogeneity of Variance test indicated homogeneous variances. Thus, a paired samples *t*‐test was used to assess overall differences between original and rotated dose distributions. Data were presented as mean ± standard deviation (minimum‐maximum), with statistical significance defined as *P* < 0.05.

## 3 RESULTS

### 3.1 Effect of rotational errors on left‐sided breast cancer patients

#### 3.1.1 Dosimetric effect of rotational errors on left‐sided breast cancer patients

Table 3A (for left‐sided) and Table 3B (for right‐sided) present a comparison of dose parameters between the original and rotated dose distributions for the three left‐sided treatment cohorts (WBRT_left4005_, WBRT_left5000_, PMRT_left5000_). The introduction of rotational errors resulted in statistically significant changes (*p* < 0.05) in D95 for PTV_sc_, mean heart dose, mean lung dose, mean contralateral breast dose, and LAD_V40_ (except for LAD_V40_ in the WBRT_left4005_ cohort, *p* = 0.058).

**TABLE 3A:** Comparison of original dose and rotated dose in left‐sided breast cancer.

		Original dose	Rotated dose	*p* values*
WBRT_left4005_				
	PTV_breast_:D95 (cGy)	4017.45 ± 22.72 (3979.33–4053.72)	3977.91 ± 46.06 (3709.45–4079)	0.000
	Heart: mean (cGy)	234.07 ± 80.89 (136.7–362.41)	238.27 ± 87.91 (110.23–452.63)	0.000
	Lung_L: mean (cGy)	662.15 ± 88.11 (527.71–776.80)	665.91 ± 93.57 (502.28–880.70)	0.000
	Controlateral breast: mean (cGy)	103.98 ± 42.29 (56.69–202.13)	110.76 ± 58.25 (43.00–346.73)	0.008
	LAD_V40_ (%)	0.26 ± 0.56 (0.00–1.86)	0.41 ± 1.12 (0.00–8.44)	0.058
WBRT_left5000_				
	PTV_sc_: D95 (cGy)	5014.55 ± 36.79 (4949.9–5061.37)	4832.25 ± 240.31 (3402.84–5130.09)	0.001
	PTV_breast_: D95 (cGy)	5023.66 ± 30.39 (4946.44–5051.33)	4959.69 ± 100.83 (4234.5–5073.65)	0.000
	Heart: mean (cGy)	398.48 ± 47.25 (327.54–466.67)	411.52 ± 95.85 (203.01–749.11)	0.000
	Lung_L: mean (cGy)	1133.87 ± 139.31 (881.53–1286.43)	1139.16 ± 151.1 (758.12–1489.22)	0.000
	Controlateral breast: mean (cGy)	232.56 ± 140.35 (49.52–481.67)	247.55 ± 164.57 (40.68–720.89)	0.001
	LAD_V40_ (%)	10.39 ± 4.92 (0.14–17.76)	13.76 ± 13.76 (0.00–62.62)	0.001
PMRT_left5000_				
	PTV_sc_:D95 (cGy)	5013.25 ± 29.42 (4948.91–5056)	4702.28 ± 308.13 (2441.12–5061.63)	0.000
	PTV_cw_: D95 (cGy)	5005.32 ± 21.1 (4961.56–5058.38)	4750.27 ± 285.09 (2778.17–5058.38)	0.000
	Heart: mean (cGy)	391.26 ± 62.93 (253.11–482.58)	405.26 ± 104.86 (137.14–818.64)	0.000
	Lung_L:mean (cGy)	1295.85 ± 81.1 (1052.02–1463.31)	1299.86 ± 104.88 (852.39–1656.11)	0.000
	Controlateral breast:mean (cGy)	345 ± 109.83 (81.64–481.67)	359.49 ± 141.81 (64.22–723.31)	0.000
	LAD_V40_ (%)	5.79 ± 4.55 (0.00–15.06)	8.93 ± 11.37 (0.00–72.89)	0.000

*Note*: WBRT_left4005_ and WBRT_left5000_ indicate prescribed dose of 4005 cGy and 5000 cGy in left‐sided WBRT. PMRT_left5000_ indicates a prescribed dose of 5000 cGy in left‐sided PMRT.

**TABLE 3B:** Comparison of original dose and rotated dose in right‐sided breast cancer.

		Original dose	Rotated dose	P values^*^
WBRT_right4005_				
	PTV_breast_:D95 (cGy)	4018.55 ± 21.57 (3976.41–4050.63)	3979.95 ± 36.61 (3846.13–4071.14)	0.000
	Heart:mean (cGy)	106.3 ± 18.26 (78.08–133.89)	107.65 ± 21.03 (71.18–167.99)	0.000
	Lung_R:mean (cGy)	640.26 ± 83.45 (520.18–789.24)	642.82 ± 83.35 (497.47–828.53)	0.000
	Controlateral breast:mean (cGy)	134.23 ± 58.75 (81.01–268.29)	143.32 ± 77.49 (68.51–464.86)	0.004
WBRT_right5000_				
	PTV_sc_:D95 (cGy)	5018.45 ± 38.01 (4938.3–5067.72)	4859.19 ± 233.57 (3351.36–5098.78)	0.002
	PTV_breast_: D95 (cGy)	5028.35 ± 16.87 (4999.45–5052.60)	4934.29 ± 154.19 (3571.75–5063.89)	0.000
	Heart:mean (cGy)	131.66 ± 28.4 (83.43–163.91)	135.44 ± 37.42 (75.95–294.99)	0.006
	Lung_R:mean (cGy)	1198.27 ± 79.7 (1078.46–1317.58)	1203.60 ± 111.42 (932.6–1512.68)	0.000
	Controlateral breast:mean (cGy)	166.31 ± 60.99 (83.52–251.54)	176.69 ± 88.46 (68.29–569.10)	0.010
PMRT_right5000_				
	PTV_sc_:D95 (cGy)	5028.76 ± 29.47 (4973.53–5074.28)	4719.89 ± 353.08 (2055.99–5121.91)	0.000
	PTV_cw_: D95 (cGy)	5009.89 ± 27.68 (4947.52–5067.12)	4838.44 ± 190.94 (3643.15–5067.12)	0.000
	Heart:mean (cGy)	238.26 ± 80.85 (103.93–399.19)	242.38 ± 87.04 (90.97–506.25)	0.000
	Lung_R:mean (cGy)	1325.14 ± 40.72 (1229.52–1409.13)	1328.5 ± 68.33 (1114.82–1607.79)	0.000
	Controlateral breast:mean (cGy)	346.30 ± 94.77 (132.96–494.64)	358.16 ± 126.34 (104.11–769.71)	0.000

*Note*: WBRT_right4005_ and WBRT_right5000_ indicate prescribed dose of 4005 cGy and 5000 cGy in right‐sided WBRT. PMRT_right5000_ indicates a prescribed dose of 5000 cGy in right‐sided PMRT.

The maximum dose uncertainty (reflecting the worst‐case degradation) for PTV coverage was observed in the PMRT_left5000_ cohort, with a 51.17% reduction in PTV_sc_:D95 and 44.75% in PTV_cw_:D95. In the WBRT_left5000_ cohort, the maximum dose uncertainty was 31.75% for PTV_sc_:D95 and 14.39% for PTV_breast_:D95. For the WBRT_left4005_ cohort, the maximum change in PTV_breast_:D95 was 7.37%.

For OARs, the mean heart dose increased by an average of 1.84% (WBRT_left4005_), 3.38% (WBRT_left5000_), and 3.64% (PMRT_left5000_) in the rotated dose. The mean dose to the ipsilateral lung showed minimal average changes (0.56%, 0.48%, and 0.31%, respectively). The mean dose to the contralateral breast increased by an average of 5.80%, 7.13%, and 4.14% across the cohorts. LAD_V40_ increased by 0.41%, 13.76%, and 8.93% in the respective cohorts.

#### 3.1.2 Radiobiological effect of rotational errors on left‐sided breast cancer patients

**FIGURE 3:**
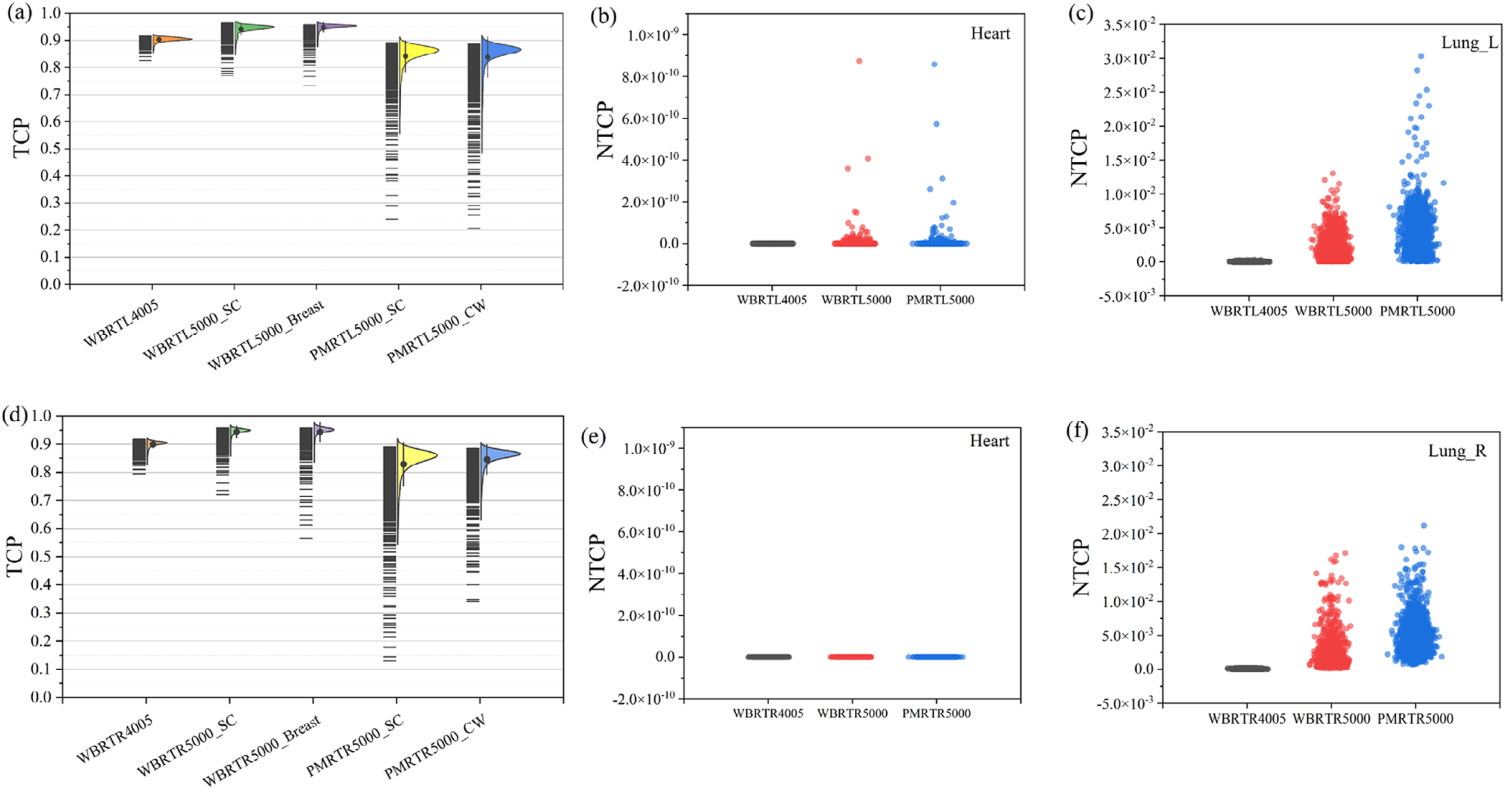
TCP and NTCP in left‐sided and right‐sided breast cancer patients. (a) TCP for PTV with different prescribed dose (left‐sided), (b) NTCP for the heart (left‐sided), (c) NTCP for the lung (left‐sided), (d) TCP for PTV with different prescribed dose (right‐sided), (e) NTCP for the heart (right‐sided), (f) NTCP for the lung (right‐sided).

The radiobiological impact of rotational errors is illustrated in Figure 3a‐c, showing the TCP and NTCP for the three left‐sided cohorts. In the WBRT_left4005_ cohort, TCP for PTV_breast_ remained high (0.90‐0.91) but dropped to a minimum of 0.83 in the worst‐case scenarios. NTCP for the heart increased from a baseline of 9.66 × 10^−16^ to a maximum of 3.53 × 10^−13^, and NTCP for the lung from 5.33 × 10^−5^ to 3.18 × 10^−4^. For WBRT_left5000_, TCP for PTV_sc_ and PTV_breast_ were mostly 0.95–0.96 but fell to 0.77 and 0.73, respectively, in some rotations. NTCP for the heart rose from 6.05 × 10^−14^ to 8.74 × 10^−10^, and NTCP for the lung from 1.67 × 10^−3^ to 1.30×10^−2^. In the PMRT_left5000_ cohort, TCP for PTV_sc_ and PTV_cw_ were 0.86–0.88 in most cases but dropped as low as 0.24 and 0.21. NTCP for the heart increased from 8.49 × 10^−16^ to 8.58 × 10^−10^, and NTCP for the lung from 3.54 × 10^−3^ to 3.03 × 10^−2^.

### 3.2 Effect of rotational errors on right‐sided breast cancer patients

#### 3.2.1 Dosimetric effect of rotational errors on right‐sided breast cancer patients

Table 3B (right‐sided) compares dose parameters for the three right‐sided cohorts. Rotational errors led to statistically significant changes (*p* < 0.05) in all evaluated parameters (D95 for PTV_,_ mean heart dose, mean lung dose, and mean contralateral breast dose).

The maximum dose uncertainty for PTV coverage was most pronounced in the PMRT_right5000_ cohort, with a 59.43% reduction in PTV_sc_:D95 and 27.80% in PTV_cw_:D95. In the WBRT_right5000_ cohort, the maximum dose uncertainty was 32.68% for PTV_sc_:D95 and 28.56% for PTV_breast_:D95. For the WBRT_right4005_ cohort, the maximum change in PTV_breast_:D95 was 3.97%.

For OARs, the mean heart dose increased by an average of 1.25% (WBRT_right4005_), 2.6% (WBRT_right5000_), and 1.72% (PMRT_right5000_). The mean dose to the ipsilateral lung showed average increases of 0.42%, 0.45%, and 0.25%, respectively. The mean dose to the contralateral breast increased by an average of 6.04%, 5.28%, and 3.21%.

#### 3.2.2 Radiobiological effect of rotational errors on right‐sided breast cancer patients

Figure 3d‐f displays the TCP and NTCP for the right‐sided cohorts. In the WBRT_right4005_ cohort, TCP for PTV_breast_ was primarily 0.91 (minimum 0.80). NTCP for heart remained at zero, while NTCP for lung increased from 5.34 × 10^−5^ to 2.03 × 10^−4^. For WBRT_right5000_, TCP for PTV_sc_ and PTV_breast_ were mainly 0.95–0.96 but dropped to 0.72 and 0.57. NTCP for the heart remained zero, and NTCP for the lung increased from 1.64 × 10^−3^ to 1.71 × 10^−2^. In the PMRT_right5000_ cohort, TCP for PTV_sc_ and PTV_cw_ were 0.86–0.89 in most cases but fell to 0.13 and 0.34 in the worst scenarios. NTCP for the heart remained zero, and NTCP for the lung increased from 3.99 × 10^−3^ to 2.12 × 10^−2^.

### 3.3 Distribution of rotational scenarios and ratios of the ROIs that exceeded the dose limits

**FIGURE 4:**
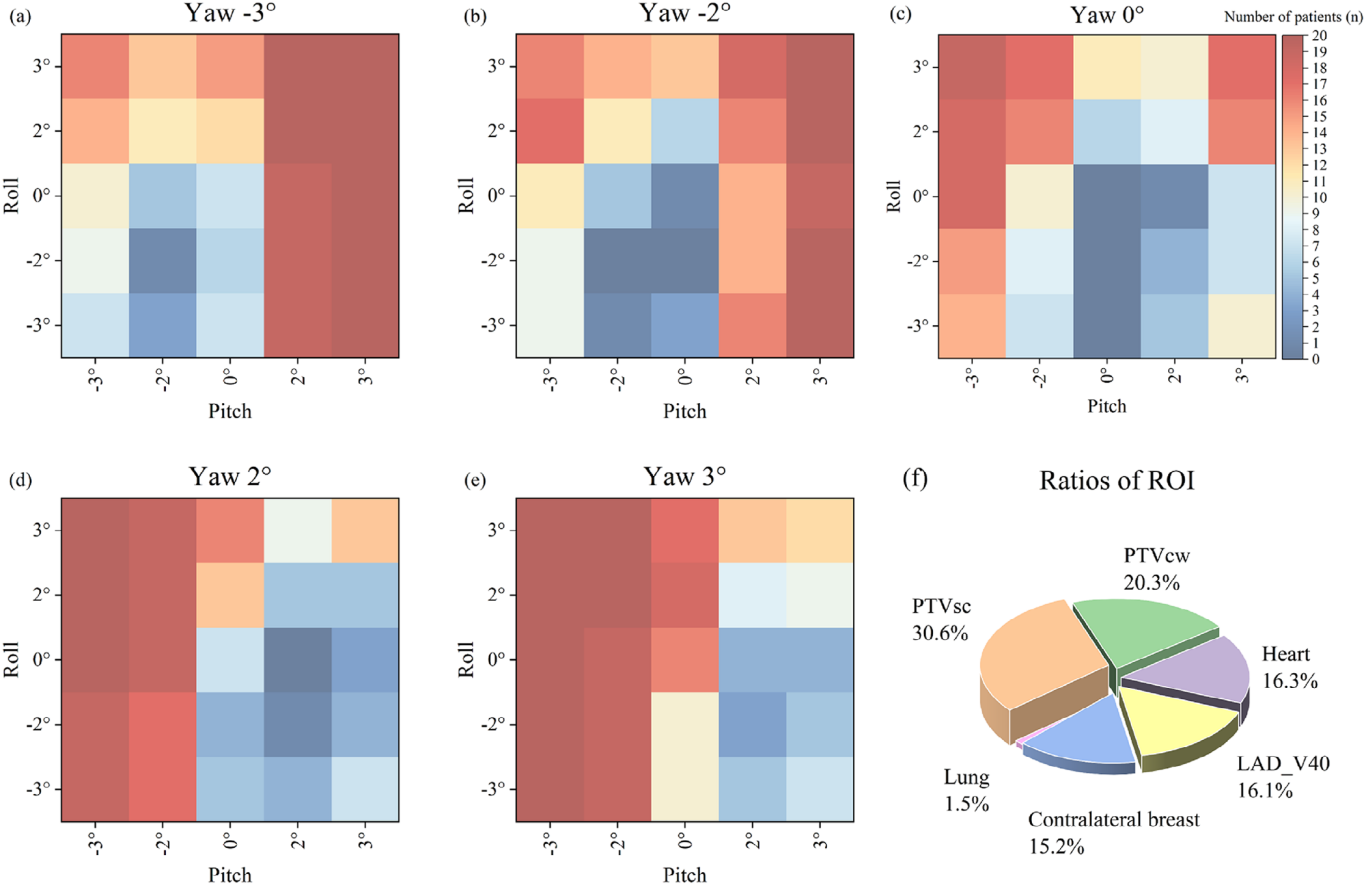
Distribution of rotational scenarios and ratios of the ROIs that exceeded the dose limits in PMRT_left5000_. (a) Distribution of pitch and roll when yaw is −3°, (b) Distribution of pitch and roll when yaw is −2°, (c) Distribution of pitch and roll when yaw is 0°, (d) Distribution of pitch and roll when yaw is 2°, (e) Distribution of pitch and roll when yaw is 3°, (f) Ratios of the ROIs that exceeded the dose limits.

The frequency distributions of rotational scenarios that exceeded the dose limits are shown in Figure 4 (for left‐sided) and Figures S1 and S2. The color intensity indicates the frequency of rotational scenarios exceeding dose limits. In the WBRT_left4005_ cohort, rotational errors rarely caused dose limit exceedances. However, in the WBRT_left5000_ and PMRT_left5000_ cohort, single‐axis rotations that exceeded dose limits were predominantly negative pitch, positive roll, and positive yaw. For multi‐axis rotations (≥2 axes), even small roll deviations could lead to significant dose shifts, particularly when yaw and pitch are rotated in opposite directions. In the WBRT_left5000_ cohort, rotational errors most frequently affected LAD_V40_ (33.5% of rotational scenarios) and PTV_sc_ (26.1%). In the PMRT_left5000_ cohort, PTV_sc_ (30.6%) and PTV_cw_ (20.3%) were the most affected structures.

The frequency distributions for right‐sided cases are provided in Figures S3, S4, and S5. The WBRT_right4005_ cohort had virtually no dose limit exceedances. In the WBRT_right5000_ and PMRT_right5000_ cohorts, single‐axis rotations leading to exceedances were mainly negative pitch, roll, and yaw. For multi‐axis rotations, small roll deviations caused dose shifts when yaw and pitch rotated in the same direction. In the WBRT_right5000_ cohort, rotational errors most frequently affected PTV_sc_ (61.9%) and PTV_breast_ (34.8%). In the PMRT_right5000_ cohort, the most affected structures were PTV_sc_ (49.1%) and PTV_cw_ (22.1%).

## 4 DISCUSSION

Although several previous studies^10^, ^33^, ^34^ have investigated the dosimetric deviations due to rotational errors, they primarily focused on the effects of single‐axis or larger rotational errors. In this study, we were the first to use RaySearch to randomly simulate rotational errors within the range of ± 3° across the pitch, roll, and yaw axes (124 scenarios per patient, with 80 patients total). Few prior studies have systematically simulated such a wide range of rotational scenarios. The findings reveal that pitch and yaw are the main contributors to dose deviations. For left‐sided breast cancer patients, when yaw rotates in the opposite direction of pitch, even minor roll‐axis rotations can induce dose deviations. Conversely, in right‐sided breast cancer cases, simultaneous rotations of yaw and pitch in the same direction enhance the probability of dose deviations, aligning with the results reported by Fu et al.^10^ Notably, pitch‐direction errors led to more pronounced dose discrepancies.

Rotational errors in the WBRT_left4005_ and WBRT_right4005_ cohorts had no effect on dose deviations to the target and OARs (as shown in Figures S2 and S5). Moreover, in most scenarios, the PTV_breast_ showed no substantial dose uncertainty (defined as a 5% dose discrepancy). This is likely because only the breast was irradiated in these scenarios. The hemispherical shape of the breast renders it less vulnerable to rotational errors.^35^, ^36^ In contrast, the PTV_sc_ showed higher sensitivity to rotational errors in the WBRT_left5000_, PMRT_left5000_, WBRT_right5000_, and PMRT_right5000_ cohorts, with observed reductions in coverage of 28.6%, 36.9%, 46.8%, and 64.2%, respectively. Sauer et al.^33^ also demonstrated that sensitivity was particularly high when supraclavicular lymph nodes were included in the irradiation field, with relative PTV coverage decreasing to 87% during large rotation. Fu et al.^10^ conducted a phantom study and found that when the pitch rotations were −2.5° and 2.5° and roll and yaw were both 3°, the reductions in the PTV:V_50 Gy_ were 20.07% and 29.58% of the original values, respectively. Our previous research^37^ has also shown that rotations greater than 2° caused a maximum dose difference of 20.92% in PTV_sc_. To mitigate this effect, it is recommended that therapists improve immobilization of the supraclavicular lymph nodes and pay more attention to it during image‐guided registration. The heart and LAD_V40_ were more significantly impacted by rotation in a higher proportion of left‐sided breast cancer patients. This effect was not observed in right‐sided breast cancer, consistent with previous studies^34^, ^38^ showing that the heart and LAD are more vulnerable to rotational effects than the lung. Rotational errors also increased the dose to the contralateral breast, though the effect remains modest. Thus, in left‐sided breast radiotherapy, the PTV_sc_ and heart (especially the LAD) should be prioritized for image‐guided registration and setup errors correction. For right‐sided breast radiotherapy, greater emphasis should be placed on the PTV_sc_ and PTV_cw_.

This study assessed the effect of rotational errors on radiobiology using the linear‐quadratic Poisson model and the Lyman‐Kutcher‐Burman model to calculate the TCP and the NTCP for the heart and lung across 124 rotational scenarios. Figure 3 shows that TCP for breast‐conserving treatment regimens primarily ranged from 0.90 to 0.91 (at a prescription dose of 4005 cGy) and from 0.95 to 0.96 (at 5000 cGy). In contrast, the PMRT cohort exhibited TCP between 0.86 and 0.88, consistent with previous studies.^29^, ^39^, ^40^ However, rotational errors induced maximum TCP reductions of 0.80 in PTV_breast_(WBRT_4005_), 0.13 in PTV_sc_ (PMRT_5000_), and 0.21 in PTV_cw_ (PMRT_5000_)—indicating that rotation more potently reduces TCP in resected patients. In this study, pericarditis^41^ and pneumonitis served as clinical endpoints for the heart and lung, respectively, with values consistent with calculations by Balasubramanian et al.^29^ Notably, the calculated heart NTCP for right‐sided breast cases was zero, as the heart typically receives lower radiation dose than left‐sided cases, yielding a negligible risk of radiation‐induced damage.^42^, ^43^ Conversely, rotational errors elevated heart and lung NTCP in WBRT_4005_, WBRT_5000_, and PMRT_5000_ cohorts to maximum values of 3.53 × 10^−13^, 8.74 × 10^−10^, 8.58 × 10^−10^ (heart) and 3.18 × 10^−4^, 1.71 × 10^−2^, 3.03 × 10^−2^ (lung), respectively—confirming that rotational errors increase the NTCP for OARs. We used widely accepted parameters from linear‐quadratic Poisson model and the Lyman‐Kutcher‐Burman model to ensure clinical relevance.^29^, ^30^, ^31^ The absolute values of TCP and NTCP depend on the model parameters (e.g., α/β ratios), but the relative trends across rotation scenarios remain robust.

Our study demonstrates that rotational errors, particularly in pitch and yaw, can significantly reduce the delivered dose to the target. Currently, the 6‐DOF couch is an important device for addressing these errors. Gevaert et al.^18^ found that the 6‐DOF couch increased the 80% prescribed dose from 95% to 100% for intracranial lesions compared to the 4‐DOF couch. In Schreibmann et al.’s study,^44^ it was demonstrated that correcting roll = 1.65° and pitch = 1.23° increased dose coverage from 49.1% to 57.2% for highly irregular tumors at the 10 Gy dose level. However, for accelerators not equipped with 6‐DOF capabilities, repositioning is a viable solution. In fact, a reasonable PTV margin can compensate for insufficient target dose coverage caused by setup errors.^45^ Zhang et al.^46^ demonstrated that a CTV‐to‐PTV margin of 6 mm was sufficient to take into account six‐dimensional set‐up errors in most patients with cervical cancer. Zeng et al.^47^ concluded that using the ITV‐to‐PTV margin of 4.0 mm (LR), 7.0 mm (SI), and 4.0 mm (AP) can ensure the target dose coverage while maintaining normal tissue dose constraints at an acceptable level for liver cancer.

Previous studies^10^, ^24^, ^34^, ^38^ tended to focus on uncertainties related to translational errors, considered only limited rotational scenarios, or lacked comprehensive modeling of TCP/NTCP. In contrast, the present study presents a rigorous and systematic analysis that accounts for rotational directions, tumor laterality, and impacts on target coverage and OARs sparing. Our study quantifies not only dosimetric deviations but also their radiobiological consequences. We also identify the most critical rotational axes and rotational combinations based on cancer laterality (right vs. left) and treatment type (WBRT vs. PMRT). Overall, this work is robust and provides important evidence for clinical practice. While our in silico simulation approach yields valuable insights, several limitations should be considered. Most importantly, some patient subgroups were modest in size (e.g., *n* = 10 for WBRT cohort), which may limit the statistical power for specific comparisons. Although small subgroup sizes are common in simulation studies, this limitation warrants caution when interpreting the results of these specific analyses. Second, 1° rotations were not included in this study, which hinders our ability to determine the impact of small rotations. Additionally, the range of simulated rotational magnitudes was broad, which precludes the definition of a precise, clinically acceptable rotational threshold. Third, we did not account for variables such as treatment technique and patient anatomy, which may influence how rotational errors affect dose distributions, even though Heikkilä et al.^34^ have compared the effects of rotational setup errors in volumetric modulated arc therapy and field‐in‐field treatment for left‐sided breast cancer. In future studies, we will integrate deep learning to better assess the impact of rotational magnitude, with the aim of defining precise clinical thresholds for image‐guided setup corrections and extending these findings to other tumor sites.

## 5 CONCLUSION

This study provides quantitative evidence demonstrating that rotational errors, particularly in pitch and yaw, induce significant dosimetric deviations. These errors compromise tumor control probability and increase normal tissue complication probability. Our findings identify which rotational axes require correction during multi‐axis rotations and provide recommendations for prioritizing the targets versus organs at risk during image‐guided registration. Implementing these corrections and prioritization strategies is crucial for optimizing the therapeutic efficacy of breast radiotherapy.

## AUTHOR CONTRIBUTIONS

Denghong Liu designed the study, collected data, analyzed data, and drafted the manuscript. Quan Zhong designed the study and analyzed data. Ya Wang collected data. Renming Zhong designed the study, revised, and finally approved the manuscript. All authors have read and confirmed the final manuscript.

## CONFLICT OF INTEREST STATEMENT

The authors declare that they have no competing interests.

## ETHICS STATEMENT

This study was approved by the Ethics Committee of West China School of Medicine, Sichuan University (Approval No. 2025227).

## Supporting information



Supporting Information

## Data Availability

The datasets used and/or analyzed during the current study are available from the corresponding author on reasonable request.
