# Supplementary material for: The dosimetric and radiobiological effects of rotational errors in breast cancer radiotherapy
Source: J Appl Clin Med Phys. 2025 Oct 15;26(10):e70303. doi: 10.1002/acm2.70303 (PMC12521787; doi:10.1002/acm2.70303)
Supplement: Supplementary file 1 — Supporting Information [file ACM2-26-e70303-s001.docx]

**
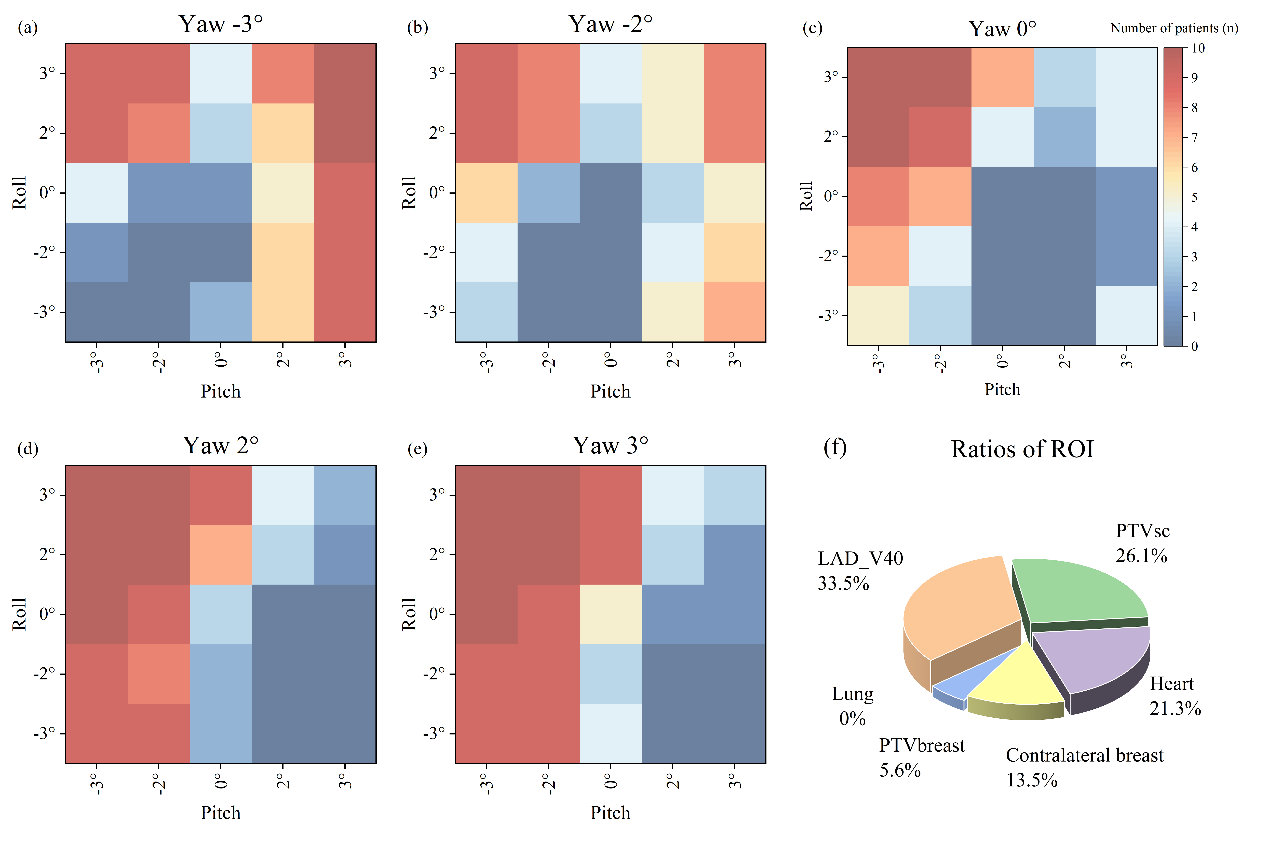
**

**Supplementary Figure S1** Distribution of rotational scenarios and ratios of the ROIs that exceeded the dose limits in WBRT_left5000_. (a) Distribution of pitch and roll when yaw is -3°, (b) Distribution of pitch and roll when yaw is -2°, (c) Distribution of pitch and roll when yaw is 0°, (d) Distribution of pitch and roll when yaw is 2°, (e) Distribution of pitch and roll when yaw is 3°, (f) Ratios of the ROIs that exceeded the dose limits.


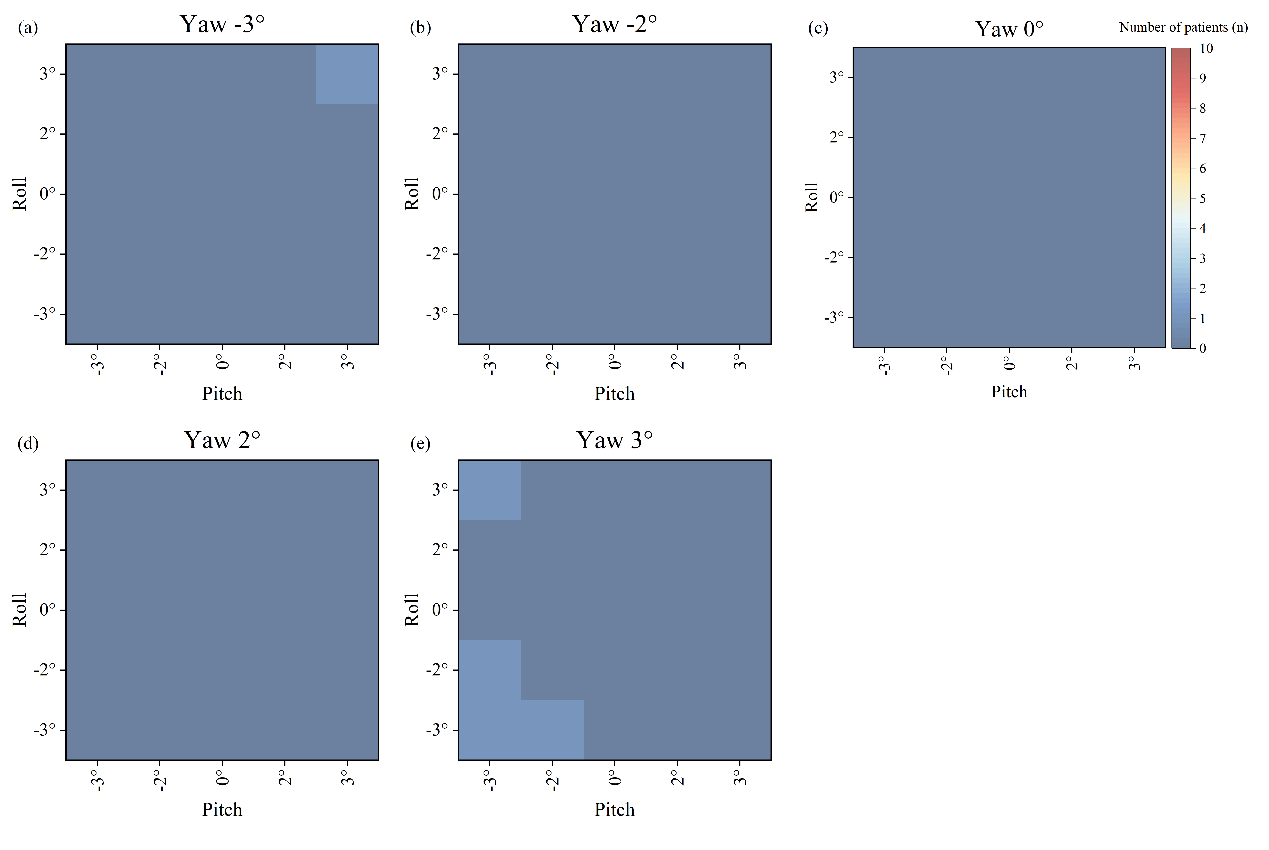


**Supplementary Figure S2** Distribution of rotational scenarios that exceeded the dose limits in WBRT_left4005_. (a) Distribution of pitch and roll when yaw is -3°, (b) Distribution of pitch and roll when yaw is -2°, (c) Distribution of pitch and roll when yaw is 0°, (d) Distribution of pitch and roll when yaw is 2°, (e) Distribution of pitch and roll when yaw is 3°.


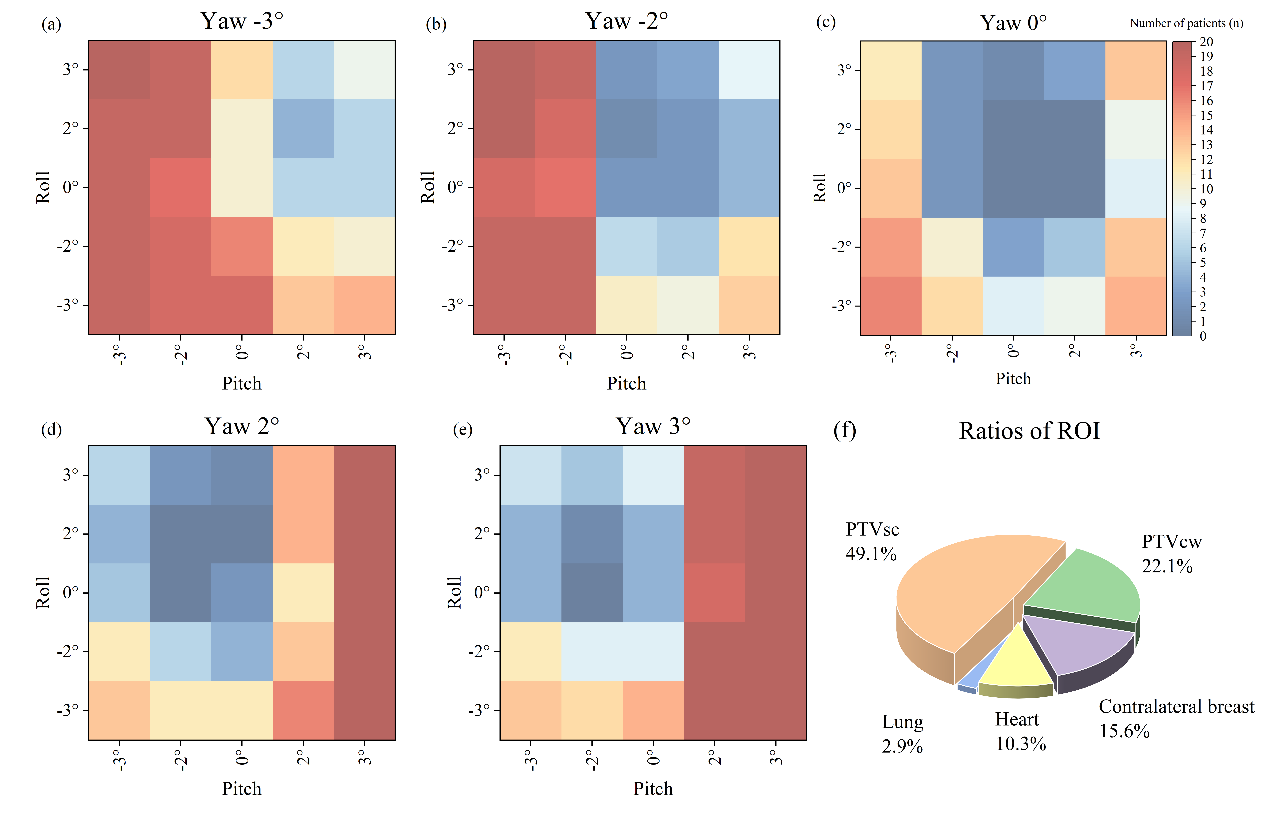


**Supplementary Figure S3** Distribution of rotational scenarios and ratios of the ROIs that exceeded the dose limits in PMRT_right5000_. (a) Distribution of pitch and roll when yaw is -3°, (b) Distribution of pitch and roll when yaw is -2°, (c) Distribution of pitch and roll when yaw is 0°, (d) Distribution of pitch and roll when yaw is 2°, (e) Distribution of pitch and roll when yaw is 3°, (f) Ratios of the ROIs that exceeded the dose limits.


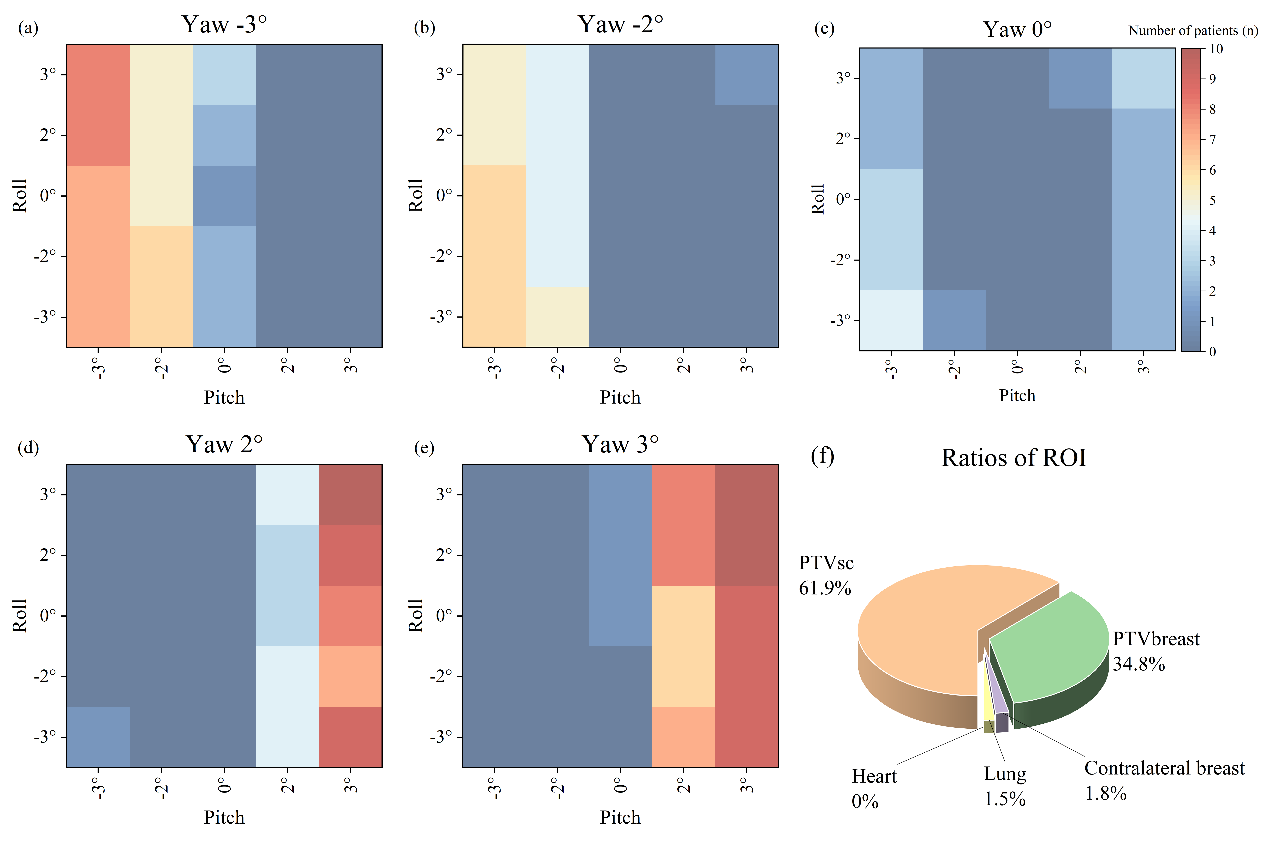


**Supplementary Figure S4** Distribution of rotational scenarios and ratios of the ROIs that exceeded the dose limits in WBRT_right5000_. (a) Distribution of pitch and roll when yaw is -3°, (b) Distribution of pitch and roll when yaw is -2°, (c) Distribution of pitch and roll when yaw is 0°, (d) Distribution of pitch and roll when yaw is 2°, (e) Distribution of pitch and roll when yaw is 3°, (f) Ratios of the ROIs that exceeded the dose limits.


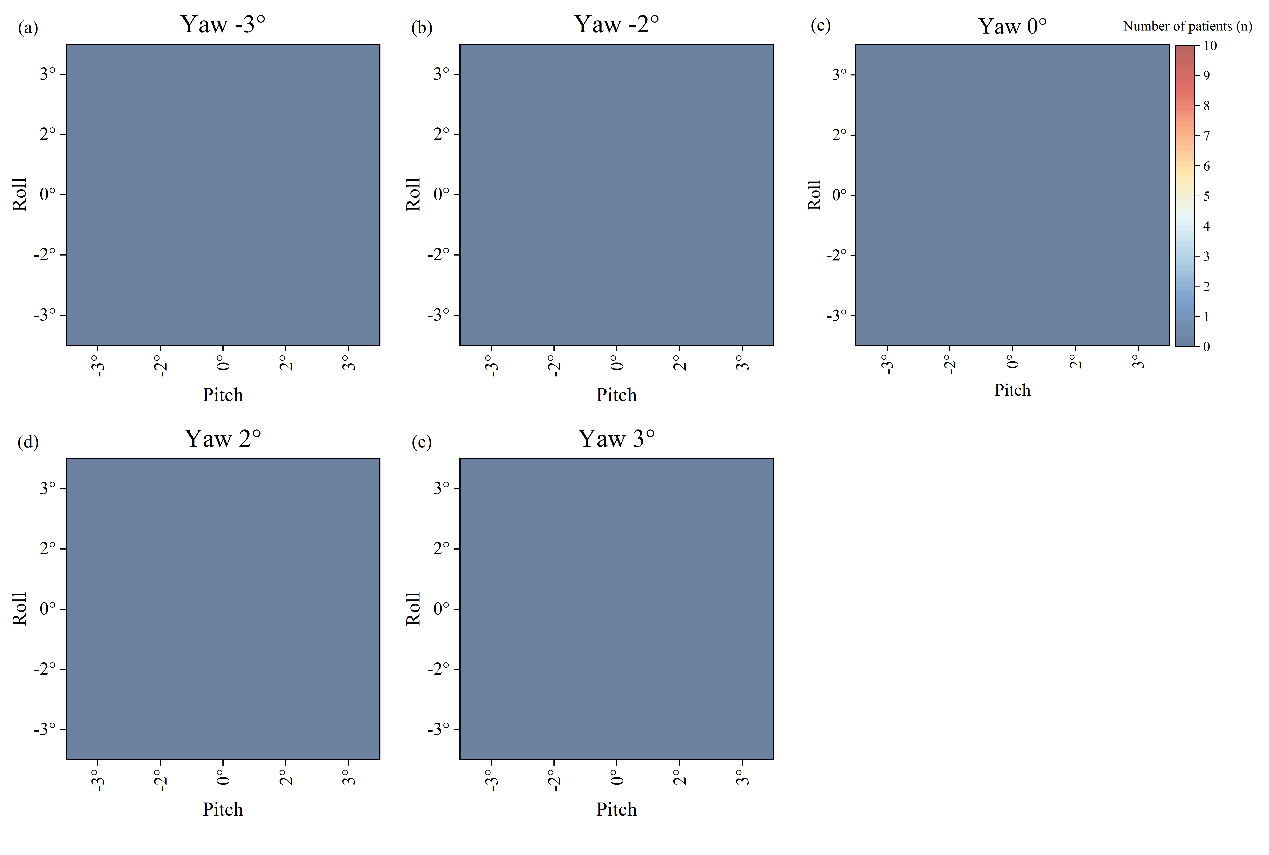


**Supplementary Figure S5**Distribution of rotational scenarios that exceeded the dose limits in WBRT_right4005_. (a) Distribution of pitch and roll when yaw is -3°, (b) Distribution of pitch and roll when yaw is -2°, (c) Distribution of pitch and roll when yaw is 0°, (d) Distribution of pitch and roll when yaw is 2°, (e) Distribution of pitch and roll when yaw is 3°.
